# Stress Echocardiography-Derived E/e’ Predicts Abnormal Exercise Hemodynamics in Heart Failure With Preserved Ejection Fraction

**DOI:** 10.3389/fphys.2019.01470

**Published:** 2019-12-03

**Authors:** Zheng-Wei Chen, Chen-Yu Huang, Jen-Fang Cheng, Ssu-Yuan Chen, Lian-Yu Lin, Cho-Kai Wu

**Affiliations:** ^1^Division of Cardiology, Department of Internal Medicine, National Taiwan University College of Medicine and Hospital, Taipei, Taiwan; ^2^Division of Cardiology, Department of Internal Medicine, National Taiwan University College of Medicine and Hospital, Yun-Lin Branch, Yun-Lin, Taiwan; ^3^Division of Cardiology, Department of Internal Medicine, Kinmen Hospital, Ministry of Health and Welfare, Kinmen, Taiwan; ^4^Division of Cardiology, Department of Internal Medicine, Pingtung Hospital, Pingtung, Taiwan; ^5^Department of Physical Medicine and Rehabilitation, Fu Jen Catholic University Hospital and Fu Jen Catholic University School of Medicine, New Taipei City, Taiwan; ^6^Department of Physical Medicine and Rehabilitation, National Taiwan University Hospital and National Taiwan University College of Medicine, Taipei, Taiwan; ^7^Cardiovascular Institute, Stanford University, Stanford, CA, United States

**Keywords:** HFpEF, stress Doppler echocardiography, hemodynamics, GLS, tissue Doppler and strain echocardiography

## Abstract

**Background:**

The correlation between echocardiographic parameters and hemodynamics data in patients with heart failure with preserved ejection fraction (HFpEF) is unclear. It is important to find a non-invasive echocardiographic parameter for predicting exercise pulmonary capillary wedge pressure (PCWP).

**Aim:**

This study sought to determine the correlation between echocardiographic parameters and hemodynamics data at rest and during exercise in HFpEF patients.

**Methods and Results:**

This study was a cross-sectional cohort exploratory analysis of baseline data from the ILO-HOPE trial. A total of 34 HFpEF patients were enrolled. The average age was 70 ± 12 years, and most (74%) were women. The patients underwent invasive cardiac catheterization and expired gas analysis at rest and during exercise. Echocardiography including tissue Doppler imaging was performed, and global longitudinal strain and other novel diastolic function indexes were analyzed at rest and during exercise. At rest, no significant correlation was noted between resting PCWP and echocardiographic parameters. However, a significant correlation was observed between post-exercise PCWP and stress E/e′ (septal, lateral, and mean) ratio (*p* = 0.003, 0.031, 0.012). Moreover, post-exercise ΔPCWP showed a good correlation with stress E/e′ (septal, lateral, and mean; all *p* ≤ 0.001) and global longitudinal strain (GLS) during exercise (*p* = 0.03). After multivariate regression analysis with adjustment for possible confounding factors including age and sex, there was still a significant correlation between post-exercise ΔPCWP and E/e′ (*r* = 0.62, *p* < 0.001 for E/e′_mean_).

**Conclusion:**

Only stress echocardiography derived tissue Doppler E/e′ ratio is closely correlated with abnormal exercise hemodynamics (PCWP and post-exercise ΔPCWP) in HFpEF. This echocardiographic marker is substantially more sensitive than other novel echocardiographic parameters during exercise, and may have significant diagnostic utility for ambulatory HFpEF patients with dyspnea.

**Clinical Trial Registration:**

https://www.clinicaltrials.gov, identifier NCT03620526.

## Introduction

Heart failure with preserved ejection fraction (HFpEF) is diagnosed using three criteria: signs and symptoms of heart failure, left ventricular (LV) ejection fraction (LVEF) > 50%, and objective evidence of diastolic dysfunction including elevated levels of natriuretic peptide and echocardiographically relevant structural heart disease or abnormal diastolic parameters (Ponikowski et al., 2016). The prevalence of HFpEF is higher than that of heart failure with reduced ejection fraction (HFrEF), and increases significantly with age. HFpEF accounts for 50% of heart failure cases in the community (Dunlay et al., 2017). Physiologically, heart failure can be defined as an imbalance between the cardiac output and metabolic demands, which generally results in increased LV filling pressure (LVFP). However, the diagnosis of HFpEF is sometimes difficult owing to non-specific symptoms, non-significantly elevated natriuretic peptide, and the absence of diastolic dysfunction on resting Doppler echocardiography (Nagueh Sherif et al., 2017). Invasive cardiac catheterization for direct hemodynamic measurements can help solve the problem: elevation of the mean pulmonary capillary wedge pressure (PCWP) could be the evidence of HFpEF (Paulus et al., 2007). Further, patients with suspected early HFpEF with normal LVFP at rest can demonstrate a steep increase in PCWP during exercise in hemodynamic stress testing. The response in a stress hemodynamics study indicates whether the symptoms are of cardiac origin (Kitzman et al., 1991; Maeder et al., 2010).

Although a hemodynamics study through cardiac catheterization remains the gold standard, it is impractical to perform invasive assessments on every patient suspected to have HFpEF, especially during exercise. Previously, the most commonly measured parameter for estimating LVFP was the ratio of early mitral inflow velocity to early diastolic tissue velocity (E/e′). However, only a few studies have evaluated the correlation between exercise E/e′ and invasively measured LVFP. Burgess et al. (2006) found a correlation between E/e′ and LVFP during exercise. Talreja et al. (2007) also found that exercise E/e′ was associated with increased PCWP (> 20 mmHg). Moreover, an abnormal response was defined as exercise E/e′ > 15 on Doppler stress echocardiography based on further outcome studies (Holland et al., 2010; Shim et al., 2011). However, some recent studies suggested that E/e′ does not reflect the LVFP increase during exercise (Maeder et al., 2010; Choi et al., 2016). Therefore, whether non-invasive measurement of E/e′ with stress Doppler echocardiography can represent increased LVFP remains unclear. In this study, we analyzed all patients from the ILO-HOPE trial to determine the relationship between echocardiographic parameters, including traditional tissue Doppler and novel strain parameters, and hemodynamics data at rest and during exercise. We aimed to find applicable echocardiographic parameters to predict abnormal exercise hemodynamics and validate the role of stress echocardiography, which may refine the diagnosis of early HFpEF.

## Materials and Methods

### Study Participants and Study Design

The study is a subgroup analysis of ILO-HOPE trial to determine the association between echocardiographic parameters and hemodynamics data. ILO-HOPE is a prospective, randomized, double-blind, placebo-controlled trial conducted to evaluate the efficacy of iloprost inhalation in improving exercise hemodynamics in HFpEF patients. However, we performed the analysis before iloprost inhalation to avoid the interference. All the patients were enrolled from cardiovascular outpatient clinics with high suspicion for HFpEF. According to the 2016 European Society of Cardiology heart failure guidelines, the American Heart Association, and our previous studies (Wu et al., 2010, 2011, 2015, 2017; Ponikowski et al., 2016), HFpEF is diagnosed according to the following criteria: (i) presence of typical symptoms and signs of heart failure, (ii) LVEF > 50%, (iii) elevated N-terminal pro-B-type natriuretic peptide (NT-proBNP) level (at least > 125 pg/mL), and (iv) echocardiographic structural [left atrial volume index > 34 mL/m^2^ or LV mass index ≥ 115 g/m^2^ (men) and ≥ 95 g/m^2^ (women)] or functional [E/e′ ≥ 13 and mean e′ (septal and lateral wall) < 9 cm/s] changes. After confirming the diagnosis of HFpEF, subjects were hospitalized for cardiac catheterization (left heart for coronary artery evaluation and right heart for hemodynamics data acquisition) and subsequent standardized exercise protocol. Informed consent was obtained before enrolling in the clinical trial. Patients with chronic renal failure (creatinine > 250 μmol/L), significant liver disease, significant coronary artery disease (coronary artery stenosis ≥ 70% without intervention, or a positive stress test), secondary hypertension, pericardial disease, significant valvular heart disease (> mild stenosis, > moderate regurgitation), cancer, cor pulmonale, congenital heart disease, left-to-right shunt, myocardial infarction within 60 days, high-output heart failure, long-term use of phosphodiesterase 5 inhibitors, or chronic atrial fibrillation were excluded.

In this subgroup analysis, we evaluated the correlation between echocardiographic parameters and hemodynamics data in different phase first (at rest and during exercise). We also performed correlation study between resting echocardiographic parameters and exercising hemodynamics in order to determine whether resting echocardiography can predict hemodynamic response during exercise.

### Standardized Exercise Protocol and Hemodynamics Data Acquisition

Cardiac catheterization for hemodynamics recording with simultaneous expired gas analysis was performed at rest and during supine exercise at a 20-W constant workload for 6 min on an electromagnetic braked cycle ergometer (Ergometrics ER800; Ergoline GmbH, Bitz, Germany), as previously described (Borlaug et al., 2015). Arterial and venous blood samples were obtained, and hemodynamic and expired gas data were acquired at rest and during exercise. Right heart catheterization through the right internal jugular vein was performed. The pressure kit transducers were zeroed at mid-axilla. Right atrial pressure, pulmonary artery (PA) pressure, and PCWP were recorded at end-expiration phase by using a 7-Fr Swan-Ganz catheter and high-fidelity micromanometer-tipped catheters (Biosensors International, Singapore) advanced through the lumen of a 7-Fr sheath (Terumo, Tokyo, Japan) in the right internal jugular vein. The mean right atrial pressure and PCWP were measured at mid A-wave. Arterial blood pressure (BP) was continuously measured using a 6-Fr catheter (Terumo) through the radial artery.

Oxygen uptake (VO_2_) data were obtained from expired gas analysis with a computerized breath-by-breath metabolic system (MetaMax 3B; Cortex Biophysik GmbH, Germany) and averaged from the 60 s preceding arterial and mixed venous blood sampling (Talreja et al., 2007). Ventilatory efficiency was checked using the ventilatory equivalent for carbon dioxide (VE/VCO_2_).

CO and stroke volume were calculated using the direct Fick method and heart rate data. Pulmonary vascular resistance (PVR), PA compliance (stroke volume/PA pulse pressure), and systemic vascular resistance were also obtained using standard formulas. LV systolic performance was assessed according to LV stroke work calculated using the standard formula.

### Two-Dimensional and Tissue Doppler Echocardiography

An echocardiographic ultrasound system (IE33; Philips, Andover, MA, United States) was used for echocardiographic examinations at rest and during exercise. Transthoracic echocardiographic images were acquired in the fundamental imaging mode. Each patient also underwent two-dimensional imaging, Doppler echocardiography, and tissue Doppler ultrasonography. LV dimensions and LVEF (M-mode) were measured in the parasternal long-axis view at rest according to the American Society of Echocardiography guidelines (Lang et al., 2005). Left atrial volume index was measured using the biplane area-length method (Lang et al., 2015). Early (E) and late (A) diastolic transmitral velocities and deceleration time were obtained using Doppler echocardiography at rest and during exercise. Peak early diastolic annular velocity was also measured at the septal (e′_septal_) and lateral (e′_lateral_) mitral annulus on tissue Doppler echocardiography at rest and during exercise. With respect to right heart function, the tricuspid regurgitation peak gradient, tricuspid annular plane systolic excursion (M-mode), and tricuspid annular systolic velocity were measured using echocardiography.

### Speckle Tracking

Echocardiographic images were analyzed offline with commercially available software (QLAB Software version 10, Cardiac Motion/Mechanics Quantification; Philips) for speckle tracking. The endocardium border was automatically detected after manually defining the points of the LV basal myocardium and LV apex. Manual adjustment was done if needed. Systolic global longitudinal strain (GLS) was calculated from the magnitude of peak longitudinal strain of 17 ventricular segments (acquired from apical four-chamber, three-chamber, and two-chamber views) according to the American Society of Echocardiography/European Association of Echocardiography consensus statement (Mor-Avi et al., 2011). During offline strain analysis, 10 patients were excluded due to inadequate image acquisition, especially during exercise. All strain analysis was conducted by two experienced cardiologists (Z-WC and C-YH) who were familiar with strain analysis. Intraobserver and interobserver reproducibility was evaluated in 15 randomly selected subjects. The coefficients of variation for GLS were 3.1 and 5.5% for intraobserver and interobserver reproducibility, respectively.

### Statistical Analysis

The results are expressed as mean ± standard deviation or *n* (%). Within-group differences of echocardiographic parameters and hemodynamics data between rest and exercise were assessed using paired Student’s *t*-test. Pearson’s correlation tests were performed to determine correlations between PCWP and echocardiographic parameters at rest and during exercise. The correlation between PCWP and NT-proBNP level was non-parametrically analyzed by Spearman’s correlation test. The change of PCWP from the rest to exercise state was recorded as ΔPCWP. The correlation between ΔPCWP and stress echocardiographic parameters was also checked. Significant determinants found in the Pearson’s correlation test (*p* ≤ 0.05) were then examined using multivariate linear regression with adjustment for age and sex. All statistical analyses were performed using SPSS for Windows version 25.0 (SPSS Inc., Chicago, IL, United States). A value of *p* ≤ 0.05 was considered statistically significant.

## Results

Thirty-four patients were enrolled in ILO-HOPE trial between January and August 2018. The baseline characteristics, including age, sex, body mass index, comorbidities, medications, and laboratory values, are summarized in Table 1. The average age was 70 ± 12 years, and 74% were women. Concerning comorbidities, 24 (71%) patients had hypertension, six (18%) had coronary artery disease, and 13 (38%) had diabetes. The median NT-proBNP level was 242 pg/mL.

**TABLE 1:** Baseline characteristics of HFpEF patients.

	**HFpEF (*N* = 34)**
Age, years	70 ± 12
Women (%)	25 (74)
Body mass index, kg/m^2^	26.1 ± 4.5
**Comorbidities**	
Coronary disease (%)	6 (18)
Hypertension (%)	24 (71)
Diabetes (%)	13 (38)
**Medications**	
ACEI or ARB (%)	19 (56)
Beta-blocker (%)	22 (65)
CCB (%)	11 (32)
Statin (%)	10 (29)
Diuretic (%)	15 (44)
Nitrate (%)	4 (12)
**Laboratory values**	
Hemoglobin, g/dL	12.4 ± 1.5
Creatinine, mg/dL	1.0 ± 0.7
NT-proBNP, pg/mL	242 (195)

*Values are mean ± standard deviation, median (interquartile range), or *n* (%). ACEI = angiotensin-converting enzyme inhibitor; ARB = angiotensin receptor blocker; CCB = calcium channel blocker; NT-proBNP = N-terminal pro-B-type natriuretic peptide.*

### Echocardiographic Parameters at Rest and During Exercise

Echocardiographic parameters measured at rest and during exercise are listed in Table 2A. The subjects had significantly higher mitral E velocity, higher mitral A velocity, shorter deceleration time, higher peak early diastolic annular velocity (septal or lateral mitral annulus), and higher E/e′ ratio during exercise than at rest. The mitral E/A ratio showed no significant difference between rest and exercise. From the strain echocardiography analysis, higher GLS magnitude and higher early diastolic strain rate (SR_e_) were noted in the exercise stage. In right-heart-related parameters, higher tricuspid regurgitation peak gradient and tricuspid annular systolic velocity were detected during exercise. The tricuspid annular plane systolic excursion was similar between the exercise and rest stages.

**TABLE 2A:** Rest and stress echocardiographic parameters in HFpEF patients (*N* = 34).

	**Rest**	**Stress**	***p*-value**
LVEDD, mm	47.00 ± 4.47		
LV ejection fraction, %	68.29 ± 7.83		
Left atrial volume index, mL/m^2^	34.15 ± 8.65		
Mitral E velocity, cm/s	85.64 ± 22.97	108.99 ± 31.35	**> 0.001**
Mitral A velocity, cm/s	89.96 ± 24.45	100.29 ± 28.51	**> 0.001**
Mitral E/A ratio	1.04 ± 0.57	1.18 ± 0.59	0.143
Deceleration time, ms	190.00 ± 49.48	140.29 ± 33.62	**0.006**
**Tissue Doppler echocardiography**			
e′_septal_, cm/s	6.68 ± 2.03	7.90 ± 2.57	**> 0.001**
e′_lateral_, cm/s	8.73 ± 2.55	9.80 ± 2.66	**> 0.001**
e′_mean_, cm/s	7.70 ± 2.18	8.85 ± 2.39	**> 0.001**
E/e′_septal_	13.40 ± 3.79	14.85 ± 6.31	**> 0.001**
E/e′_lateral_	10.09 ± 2.41	12.52 ± 7.97	**0.011**
E/e′_mean_	11.44 ± 2.77	13.37 ± 6.88	**0.001**
**Strain echocardiography**			
GLS,%	−17.33 ± 1.97	−18.39 ± 2.39	**0.009**
AP2 L. strain,%	−17.70 ± 1.98	−18.77 ± 2.88	**0.038**
AP3 L. strain,%	−17.23 ± 2.75	−17.88 ± 2.73	0.248
AP4 L. strain,%	−17.53 ± 2.25	−18.50 ± 2.16	**0.002**
SR_IVR_, 1/s	0.28 ± 0.11	0.30 ± 0.10	0.396
E/SR_IVR_, cm	335.76 ± 127.33	384.91 ± 158.59	0.194
SR_e_, 1/s	0.77 ± 0.18	0.98 ± 0.22	**> 0.001**
E/SR_e_, cm	111.68 ± 30.45	113.47 ± 42.54	0.779
**Right heart function parameters**			
TRPG, mmHg	27.17 ± 9.18	41.83 ± 10.84	**> 0.001**
TAPSE, cm	2.29 ± 0.45	2.76 ± 1.99	0.281
TAS′, cm/s	13.18 ± 2.92	14.80 ± 4.46	**> 0.001**

*Values are mean ± standard deviation. LVEDD = left ventricular end diastolic dimension; mitral E/A ratio = ratio of peak early (E) to peak late (A) diastolic transmitral velocities; e′septal/lateral/mean = peak early diastolic annular velocity measured at the septal/lateral mitral annulus and their mean; E/e′septal/lateral/mean = ratio of E to e′septal/lateral/mean; GLS = global longitudinal strain; AP2/AP3/AP4 L. strain = longitudinal strain in apical two-chamber/three-chamber/four-chamber view; SR_*IVR*_ = strain rate during isovolumetric relaxation; E/SR_*IVR*_ = ratio of E to SR_*IVR*_; SR_*e*_ = early diastolic strain rate; E/SR_*e*_ = ratio of E to SR_*e*_; TRPG = tricuspid regurgitation peak gradient; TAPSE = tricuspid annular plane systolic excursion; TAS′ = tricuspid annular systolic velocity. The bold font character means statistically significance (*p* < 0.05).*

### Hemodynamics Data at Rest and During Exercise

Resting and exercise hemodynamic changes were recorded (Table 2B). At rest, the subjects had elevated BP (systolic BP = 170 ± 23 mmHg, mean BP = 108 ± 13 mmHg), elevated PCWP (18 ± 7 mmHg), mildly increased PVR (1.02 ± 0.94 mmHg/L/min), and normal CO (5.3 ± 2.2 L/min). During exercise, all subjects had significantly increased heart rate, BP, PA pressure, PCWP, LV stroke work, and cardiac output. Concerning metabolic factors, both VO_2_ and CaO_2_–CvO_2_ significantly increased during exercise. However, PVR and PA compliance presented a downtrend after exercise but without statistical significance.

**TABLE 2B:** Baseline and exercise hemodynamics in HFpEF patients (N = 34).

	**Rest**	**20-W exercise**
**Vital signs**		
Heart rate, beats/min	69 ± 10	102 ± 23^†^
Systolic BP, mmHg	170 ± 23	185 ± 45^‡^
Diastolic BP, mmHg	77 ± 12	81 ± 13^‡^
Mean BP, mmHg	108 ± 13	118 ± 16^†^
**Central pressures**		
RA, mmHg	9 ± 4	15 ± 6^†^
PA systolic, mmHg	34 ± 11	55 ± 15^†^
PA mean, mmHg	22 ± 7	37 ± 11^†^
PCWP, mmHg	18 ± 7	29 ± 9^†^
**Vascular and ventricular function**		
PVR, mmHg/L/min	1.02 ± 0.94	0.96 ± 1.00
PA compliance, mL/mmHg	5.1 ± 2.8	4.2 ± 2.8
SVR, DSC	1699 ± 614	969 ± 372^†^
LVSW, g/beat	95 ± 43	113 ± 40^‡^
**Integrated function and metabolism**		
VO_2_, mL/min	218 ± 79	572 ± 131^†^
CaO_2_–CvO_2_, mL/dL	4.3 ± 0.8	6.5 ± 1.8^†^
CO, L/min	5.3 ± 2.2	9.5 ± 4.0^†^
Stroke volume, mL	78 ± 36	96 ± 37^‡^

*Values are mean ± standard deviation. ^∗^Columns show rest and exercise hemodynamics. All between-group comparisons at rest and during exercise are *p* = not significant. ^†^*p* < 0.0001 versus baseline, within-subject change. ^‡^*p* ≦ 0.05 versus baseline, within-subject change. BP = blood pressure; CaO_2_–CvO_2_ = arteriovenous O_2_ content difference; CO = cardiac output; DSC = dyne/s⋅cm^5^; LVSW = left ventricular stroke work; PA = pulmonary artery; PCWP = pulmonary capillary wedge pressure; PVR = pulmonary vascular resistance; RA = right atrial; SVR = systemic vascular resistance; VO_2_ = oxygen consumption; W = watts.*

### Correlation Between PCWP and Echocardiographic Parameters

**FIGURE 1:**
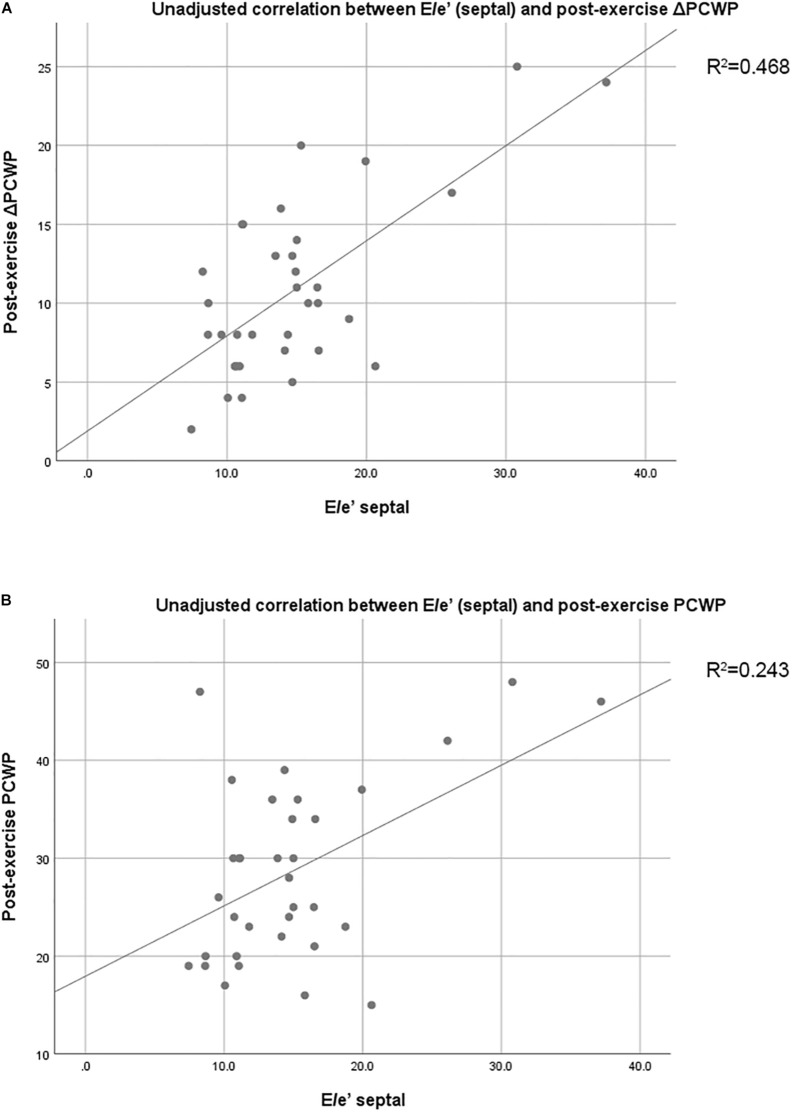
**(A)** Unadjusted correlation between E/e′_septal_ and post-exercise PCWP. **(B)** Unadjusted correlation between E/e′_septal_ and post-exercise ΔPCWP. PCWP, pulmonary capillary wedge pressure; Δ, change; E/e′_septal_, ratio of peak early diastolic transmitral velocity to peak early diastolic annular velocity measured at the septal mitral annulus.

At rest, no echocardiographic parameters, including tissue Doppler and strain echocardiography, correlated well with PCWP (Table 3A). Among the clinical parameters, only NT-proBNP showed a significant correlation with resting PCWP (*p* = 0.028) (Table 3B). During exercise, mitral E velocity, mitral E/A ratio, deceleration time, and E/e′_septal/lateral/mean_ revealed significant correlations with exercising PCWP (Table 3A). Moreover, stress echocardiographic parameters, including mitral E velocity, mitral E/A ratio, deceleration time, and E/e′_septal/lateral/mean_, showed an even better correlation with post-exercise ΔPCWP (Table 3A). GLS also showed a significant correlation (*p* = 0.03) with ΔPCWP. These significant parameters remained independent factors after multivariate linear regression analysis with adjustment for age and sex (Table 4). The correlation between exercise E/e′_septal_ and post-exercise PCWP/ΔPCWP is plotted in Figures 1A,B.

**TABLE 3A:** Correlation between rest/post-exercise PCWP/ΔPCWP and echocardiographic parameters.

	**Rest PCWP**		**Post-exercise PCWP**		**ΔPCWP**	
**Rest echocardiographic**	**Pearson correlation**	***p*-value**	**Pearson correlation**	***p*-value**	**Pearson correlation**	***p*-value**
**parameters**	**coefficient**		**coefficient**		**coefficient**	
LVEDD	0.086	0.634				
LV ejection fraction	−0.302	0.087				
Left atrial volume index	−0.093	0.608				
Mitral E velocity	0.221	0.209			0.032	0.859
Mitral E/A ratio	0.224	0.211			−0.135	0.454
Deceleration time	−0.257	0.143			−0.175	0.322
e′_septal_	0.313	0.071			−0.235	0.181
e′_lateral_	0.096	0.590			−0.148	0.404
e′_mean_	0.202	0.252			−0.196	0.266
E/e′_septal_	−0.061	0.732			0.351	**0.042**
E/e′_lateral_	0.078	0.662			0.180	0.309
E/e′_mean_	−0.004	0.981			0.274	0.117
GLS	0.186	0.384			0.189	0.378
SR_IVR_	0.025	0.907			−0.108	0.615
E/SR_IVR_	0.099	0.645			0.262	0.217
SR_e_	0.226	0.287			−0.058	0.788
E/SR_e_	0.086	0.689			0.215	0.312
TRPG	0.195	0.268			−0.061	0.732
TAPSE	−0.092	0.603			−0.075	0.674
TAS′	−0.161	0.363			−0.089	0.617
**Stress echocardiographic parameters**						
Mitral E velocity			0.469	**0.005**	0.532	**0.001**
Mitral E/A ratio			0.559	**0.001**	0.673	**<0.001**
Deceleration time			−0.380	**0.026**	−0.525	**0.001**
e′_septal_			−0.031	0.860	−0.216	0.219
e′_lateral_			−0.010	0.953	−0.252	0.151
e′_mean_			−0.023	0.898	−0.257	0.142
E/e′_septal_			0.493	**0.003**	0.684	**<0.001**
E/e′_lateral_			0.371	**0.031**	0.546	**0.001**
E/e′_mean_			0.425	**0.012**	0.620	**<0.001**
GLS			0.377	0.069	0.443	**0.030**
SR_IVR_			0.097	0.654	0.278	0.188
E/SR_IVR_			0.273	0.197	0.207	0.333
SR_e_			0.194	0.364	0.151	0.482
E/SR_e_			0.337	0.107	0.369	0.076
TRPG			0.236	0.179	0.130	0.465
TAPSE			−0.065	0.716	0.136	0.444
TAS′			−0.295	0.101	−0.267	0.139

*LVEDD = left ventricular end diastolic dimension; mitral E/A ratio = ratio of the peak early (E) to peak late (A) diastolic transmitral velocities; e′septal/lateral/mean = peak early diastolic annular velocity measured at the septal/lateral mitral annulus and their mean; E/e′septal/lateral/mean = ratio of E to e′septal/lateral/mean; GLS = global longitudinal strain; SR_*IVR*_ = strain rate during isovolumetric relaxation; E/SR_*IVR*_ = ratio of E to SR_*IVR*_; SR_*e*_ = early diastolic strain rate; E/SR_*e*_ = ratio of E to SR_*e*_; TRPG = tricuspid regurgitation peak gradient; TAPSE = tricuspid annular plane systolic excursion; TAS′ = tricuspid annular systolic velocity. The bold font character means statistically significance (*p* < 0.05).*

**TABLE 3B:** Correlation between rest/post-exercise PCWP and clinical parameters.

	**Rest PCWP**		**Post-exercise PCWP**	
**Clinical parameters**	**Pearson correlation coefficient**	***p*-value**	**Pearson correlation coefficient**	***p*-value**
Age	−0.088	0.622	−0.042	0.812
Sex	0.300	0.085	0.151	0.395
Body mass index	0.192	0.277	0.059	0.740
**Comorbidities**				
Coronary disease	0.155	0.383	0.241	0.169
Hypertension	−0.052	0.772	−0.056	0.754
Diabetes	0.263	0.133	0.180	0.307
**Medications**				
ACEI or ARB	0.190	0.282	0.218	0.216
Beta-blocker	0.149	0.401	0.118	0.505
CCB	−0.163	0.356	−0.006	0.975
Statin	0.216	0.221	0.284	0.103
Diuretic	0.022	0.900	−0.041	0.818
Nitrate	0.122	0.492	0.036	0.842
**Laboratory values**				
Hemoglobin	−0.124	0.486	−0.122	0.492
Creatinine	0.272	0.120	0.231	0.188
NT-proBNP^∗^	0.483	**0.004**	0.333	0.054

*^∗^The correlation between PCWP and NT-proBNP level was non-parametrically analyzed by Spearman’s correlation test. ACEI = angiotensin-converting enzyme inhibitor; ARB = angiotensin receptor blocker; CCB = calcium channel blocker; NT-proBNP = N-terminal pro-B-type natriuretic peptide. The bold font character means statistically significance (*p* < 0.05).*

**TABLE 4:** Multivariate regression analysis with post-exercise PCWP and ΔPCWP as the dependent variable (adjusted for age and sex) (*N* = 34).

	**Post-exercise PCWP**			**Post-exercise ΔPCWP**		
**Variable**	**β (95% CI)**	**Adjusted *R*^2^**	***p*-value**	**β (95% CI)**	**Adjusted *R*^2^**	***p*-Value**
Mitral E velocity, cm/s	0.137 (0.044–0.231)	0.195	**0.005**	0.094 (0.040–0.148)	0.261	**0.001**
Mitral E/A ratio	8.857 (3.956–13.757)	0.289	**0.001**	6.482 (3.824-9.140)	0.434	**<0.001**
Deceleration time, ms	−0.104 (−0.195 to −0.013)	0.118	**0.026**	−0.087 (−0.138 to −0.036)	0.253	**0.001**
E/e′_septal_	0.718 (0.262–1.174)	0.220	**0.003**	0.603 (0.372–0.834)	0.451	**<0.001**
E/e′_lateral_	0.427 (0.042–0.813)	0.110	**0.031**	0.381 (0.170–0.591)	0.276	**0.001**
E/e′_mean_	0.568 (0.133–1.002)	0.155	**0.012**	0.501 (0.273–0.729)	0.365	**<0.001**
GLS, %	–	–	**–**	1.147 (0.122–2.171)	0.160	**0.030**

*Mitral E/A ratio = ratio of the peak early (E) to peak late (A) diastolic transmitral velocities; e′septal/lateral/mean = peak early diastolic annular velocity measured at the septal/lateral mitral annulus and their mean; E/e′septal/lateral/mean = ratio of E to e′septal/lateral/mean; GLS = global longitudinal strain. The bold font character means statistically significance (p < 0.05).*

## Discussion

To our knowledge, this is the first study to examine and compare the correlation between LV diastolic echocardiographic parameters, including traditional tissue Doppler and novel strain analysis, and PCWP in HFpEF patients. No resting echocardiographic correlated significantly with resting PCWP, while some stress echocardiographic transmitral E wave-derived parameters (mitral E velocity, mitral E/A ratio, deceleration time, and E/e′_septal/lateral/mean_) correlated well with PCWP during exercise. We also found that exercise E/e′ correlated better with PCWP increase than post-exercise PCWP, which emphasizes the importance of diastolic stress echocardiography. Diastolic stress echocardiography was applied to detect impaired LV diastolic function reserve during exercise (Lancellotti et al., 2016). It is of value in the diagnosis of HFpEF in patients with symptoms of breathlessness and poor exercise capacity. Recent guidelines suggested that HFpEF can be diagnosed on the basis of symptoms, preserved ejection fraction, and objective evidence of echocardiographic diastolic dysfunction (Ponikowski et al., 2016). However, HFpEF symptoms often occur during exercise because LVFP can be normal at rest and only increase during exercise, which, in turn, leads to dyspnea and effort intolerance (Holland et al., 2010). Further, even with the newest recommendations for LV diastolic function evaluation, some patients are still classified as indeterminate (Nagueh et al., 2016). The recommended modality is supine bicycle, which allows Doppler recordings and diastolic function assessment throughout the test. Normal hemodynamic changes in HFpEF patients included elevations in LV end-diastolic pressure (LVEDP), PCWP, and PA systolic pressure, which occur in parallel to each other. To non-invasively estimate hemodynamic changes, it is important to validate the correlation between echocardiographic parameters and hemodynamics data, especially PCWP and LVEDP.

Among the diastolic echocardiographic parameters, E/e′ was the most established parameter that is correlated with LVFP. However, the correlation was validated and more reliable in HFrEF (Ommen et al., 2000; Ritzema et al., 2011). Moreover, the existing studies are relatively few and showed only a moderate correlation. A systematic review published in 2016 disclosed that there is insufficient evidence supporting the estimation of LVFP with E/e′, and that the diagnostic accuracy of E/e′ is limited (Sharifov et al., 2016). The pooled correlation coefficient between E/e′ and invasively measured LVFP was 0.56 (Nauta et al., 2018). Moreover, previous studies had small sample sizes and included a wide variety of cardiac diseases, which are not specific for HFpEF patients. Echocardiography and invasive hemodynamics measurements were not always performed simultaneously. Otherwise, very few studies reported the correlations between invasive hemodynamics parameters and other echocardiographic parameters. From the multicentre EACVI Euro-filling study in 2017 (Lancellotti et al., 2017), only mitral E velocity (*p* = 0.003), mitral E/A ratio (*p* = 0.01), deceleration time (*p* = 0.0005), and E/e′_lateral_ (*p* = 0.03) significantly correlated with invasive LVEDP (estimated using PCWP) in the subgroup analysis of patients with LVEF > 50%. Both E/e′_septal_ and E/e′_mean_ did not correlate well with invasive LVEDP. Further analysis showed no significant difference with regard to percentage in different cut-off of diastolic parameters (e′_septal_ < 7 cm/s, e′_lateral_ < 10 cm/s, E/e′_septal_ ≥ 15, E/e′_lateral_ ≥ 13, E/e′_mean_ ≥ 14, left atrial volume index ≥ 34 mL/m^2^, tricuspid regurgitation velocity ≥ 2.8 m/s) between LVEDP ≥ 15 and < 15 mmHg. The current study population was entirely composed of HFpEF patients. The correlation between main diastolic echocardiographic parameters and PCWP at rest was even poorer in our analysis. Although novel strain echocardiography parameters were also analyzed, the GLS, strain rate (either in isovolumetric relaxation or early diastolic phase), and ratio of mitral E velocity to strain rate all showed no significant correlation to PCWP at rest.

Although diastolic stress echocardiography may help in the diagnosis of HFpEF, the correlation between exercise E/e′ and invasively measured LVFP remains inconclusive. Some studies found a good correlation between exercise E/e′ and LVFP (Burgess et al., 2006; Talreja et al., 2007; Obokata et al., 2017) and even that exercise E/e′ was an independent predictor of outcomes (Holland et al., 2010; Shim et al., 2011; Takagi et al., 2014; Kosmala et al., 2018a, b), but some did not (Maeder et al., 2010; Choi et al., 2016). In their 2017 systematic review, Oleg et al. concluded that the evidence for the usefulness of E/e′ in estimating LVFP during exercise remains limited (Sharifov and Gupta, 2017). Our study provided comprehensive measurements of resting and exercising echocardiographic parameters, as well as simultaneous invasive hemodynamics studies at rest and exercise in our cohort of purely HFpEF patients. From our analysis, mitral E velocity, mitral E/A ratio, deceleration time, and E/e′_septal/lateral/mean_ showed a good correlation with PCWP during exercise.

It had been known that transmitral Doppler E wave is proportionate to the difference between left atrium (LA) pressure and LV diastolic pressure, which was influenced by the rate of myocardial relaxation. Otherwise, tissue Doppler e’ velocity is a measure of LV myocardial relaxation in early diastole and relatively load independent (Agmon et al., 2000). As a result, it can be inferred that transmitral E wave-derived parameters show some correlation with PCWP, and combination of E and e’ (E/e’) may be a better predictor. However, from our result, we found these transmitral E wave-derived parameters (mitral E velocity, mitral E/A ratio, deceleration time, and E/e′_septal/lateral/mean_) only correlated with PCWP significantly when exercising but not at rest. It may be speculated that the correlation between these transmitral E wave-derived parameters and PCWP only exists significantly in condition of elevated LVFP and impaired myocardial relaxation (such as HFrEF or HFpEF when exercising).

Moreover, we found that only resting E/e′_septal_ correlated with increased PCWP during exercise (Table 3A). However, in the exercise stage, echocardiographic parameters including mitral E velocity, mitral E/A ratio, deceleration time, and E/e′_septal/lateral/mean_ showed a much better correlation with ΔPCWP (Table 3A). These result indicated that the severity of diastolic dysfunction or impaired myocardial relaxation during exercise may influence the change of PCWP more rather than PCWP during exercise. Further, the significant correlations remained after multivariate regression analysis with adjustment for possible confounding factors including age and sex. Dorfs et al. (2014) demonstrated that PCWP increase was associated with increased mortality despite a normal resting PCWP. Reddy et al. (2018) also reported that increased PCWP was associated with reduced exercise capacity. Otherwise, ΔE/e′_septal/lateral/mean_ also correlated well with ΔPCWP (Supplementary Table S1). All these findings emphasize the importance of diastolic stress echocardiography. On the basis of current evidence, we recommend diastolic stress echocardiography as a diagnostic tool for patients suspected of having HFpEF, especially those with a normal or indeterminate resting diastology.

We also performed strain analysis through two-dimensional echocardiographic speckle tracking. Strain is the measurement of myocardium deformation, whereas the strain rate is the speed of myocardial deformity. In previous studies, HFpEF patients had a lower magnitude of GLS and decreased strain rate despite preserved LVEF compared with normal controls (Kraigher-Krainer et al., 2014; Tabassian et al., 2018). Moreover, GLS is associated with reduced exercise capacity in HFpEF patients (Hasselberg et al., 2015). Wang et al. (2007) showed that E/SR_IVR_ best correlated with PCWP, especially when E/e′ ranged from 8 to 15. Magoon et al. (2018) also found that E/SR_e_ had a better correlation with PCWP than E/e′_septal_ in patients undergoing coronary artery bypass grafting with preserved ejection fraction. Meanwhile, Ebrahimi et al. (2019) reported that SR_IVR_ was a better index for predicting PCWP intra-operatively in patients undergoing coronary artery bypass grafting. However, their study population all had coronary artery disease, and the authors performed the measurements after general anesthesia induction. In our study, although the novel parameter GLS significantly correlated with ΔPCWP during exercise (*p* = 0.03), other diastolic strain-based indices showed a poor correlation with PCWP, either at rest or during exercise. In summary, strain echocardiography has better sensitivity to detect subclinical impairment of systolic function or subtle diastolic dysfunction (Chen et al., 2018), E/e’ has better correlation with ΔPCWP during exercise.

At last, the BP response to exercise is an important diagnostic parameter. In healthy subjects, systolic BP rise according to the increasing workload. However, diastolic BP usually remained unchanged or decrease slightly (O’Brien et al., 2002). In baseline characteristics of HFpEF patients, we found that the diastolic pressure increased significantly after 20-W exercise (Table 2B). These suggested the HFpEF patient in our study have stiff arteries. Chantler et al. (2008a) investigated the influence of arterial system on left ventricle performance. This interaction is called arterial–ventricular coupling, which could be indexed by the ratio of effective arterial elastance to LV end-systolic elastance (E_A_/E_LV_). During exercise, E_LV_ increased disproportionately to make sure the sufficient cardiac performance to meet the needs of the body. Borlaug et al. (2006) found that HFpEF patient had a threefold smaller increase in E_LV_ during upright bicycle exercise, compared with hypertensive patients with LV hypertrophy. As a result, the change of E_A_/E_LV_ during exercise may also be blunted. Otherwise, it can be inferred that these effects have contributed to the exercise intolerance in HFpEF patients (Chantler et al., 2008b), which could be reflected by increased LVFP during exercise and subsequent abnormal stress echocardiographic parameters.

### Clinical Implication

Invasive hemodynamic measurements can help solve the confusion in diagnosing HFpEF. The mean PCWP confirms the diagnosis of HFpEF (Paulus et al., 2007), and hemodynamic stress testing could be considered in “gray cases” of patients with early HFpEF with normal filling pressure at rest. In such cases, a steep increase in PCWP during exercise is a typical hemodynamic response in HFpEF, indicating that the dyspnea on exertion is of cardiac origin (Kitzman et al., 1991). Moreover, HFpEF patients usually experience hemodynamic derangement especially during exercise, presenting as a higher LVFP (PCWP). HFpEF is an increasingly recognized cause of pulmonary hypertension due to its emerging epidemic. Some recent studies have shown that the exercise PCWP level is highly associated with the symptoms and life quality of HFpEF patients (Obokata et al., 2018), and more clinical trials have investigated exercise PCWP as a primary outcome (Borlaug et al., 2015). Theoretically, it is not possible to perform invasive exercise hemodynamic testing in every patient. Despite the increasing number of emerging diastolic function echocardiographic parameters, our study suggested exercise E/e’ to non-invasively estimate the possible hemodynamic response. By performing echocardiography during standardized exercise tests, the risk and outcomes may be predicted, consequently allowing treatment plan adjustments for HFpEF patients.

### Study Limitations

The main limitation of our study is the relatively small sample size. For this reason, some echocardiographic parameters, including strain echocardiography-derived parameters, might not correlate well with PCWP. Moreover, though some parameters correlated significantly, statistical type II error might exist. Second, this study is a subgroup analysis from ILO-HOPE trial. All patient recruitment and exclusion criteria were designed for ILO-HOPE trial. For example, the patients with chronic atrial fibrillation were excluded, and they are not uncommon in HFpEF populations. However, we believe that these selection criteria can also be applied appropriately in our subgroup analysis to evaluate the correlation between echocardiographic parameters and hemodynamics data for most HFpEF patients. Third, some medication may influence the strain analysis (especially beta-blockers), reduce preload, and alleviate LVFP (ACEI or ARB, diuretics, and nitrate). However, the improvement of hemodynamics change is parallel to echocardiographic parameter. Our main finding may not be affected. Fourth, our current study measured echocardiographic data and cardiac performance at rest and under limited levels of exercise but not maximal-effort exercises. As a result, the correlation between hemodynamics data and echocardiographic parameters was unknown at peak exercise. However, it would be difficult for patients to do peak exercises repeatedly in one single test and usually HFpEF patients perform low level of exercises in their daily life, especially the elderly. Fifth, our cross-sectional study cannot infer causality. Also, the coefficient of determination (adjusted *R*^2^) in correlation between E/e’(septal) and post-exercise ΔPCWP is only 0.468. The strength of correlation might be from few patients in the population. Further large-scale studies are required to evaluate the capacity of exercise E/e’ to predict ΔPCWP during exercise in HFpEF patients.

## Conclusion

E/e′ showed a significant correlation with both exercise PCWP and ΔPCWP even after adjustment for age and sex. Nevertheless, novel strain rate indices showed no association with PCWP and ΔPCWP, whereas GLS correlated with ΔPCWP. As exercise PCWP and ΔPCWP reflect the symptoms of HFpEF patients, exercise E/e′ may further refine the diagnosis of HFpEF. Our study results emphasize the clinical value of diastolic stress echocardiography.

## Data Availability Statement

The raw data supporting the conclusion of this manuscript will be made available by the authors, without undue reservation, to any qualified researcher.

## Ethics Statement

The study was performed in accordance with the Declaration of Helsinki and was approved by the institutional review board of the National Taiwan University Hospital (Clinical trial number: 201704075MIND). All patients provided their written informed consent prior to participation in the study.

## Author Contributions

C-KW, Z-WC, and L-YL designed the whole study, and analyzed and interpreted the data. C-KW and Z-WC wrote the manuscript. S-YC was also responsible for measurement of oxygen uptake and the computerized breath-by-breath metabolic system. C-KW and L-YL recruited the patients and were also in charge of the whole program. C-YH and Z-WC performed cardiac catheterization and echocardiography for the patients. All the authors critically reviewed the manuscript for important intellectual content.

## Conflict of Interest

The authors declare that the research was conducted in the absence of any commercial or financial relationships that could be construed as a potential conflict of interest.
