# Supplementary material for: Stress Echocardiography-Derived E/e’ Predicts Abnormal Exercise Hemodynamics in Heart Failure With Preserved Ejection Fraction
Source: Front Physiol. 2019 Dec 3;10:1470. doi: 10.3389/fphys.2019.01470 (PMC6901703; doi:10.3389/fphys.2019.01470)
Supplement: Supplementary file 1 [file Table_1.docx]

**Supplementary Data**

| Table S1. Correlation between post-exercise ΔPCWP and ΔE/e′ | | |
| --- | --- | --- |
| Echocardiographic parameters | **Pearson correlation coefficient** | **p-Value** |
| ΔE/e′_septal_ | 0.581 | **<0.001** |
| ΔE/e′_lateral_ | 0.540 | **0.001** |
| ΔE/e′_mean_ | 0.594 | **<0.001** |
| E/e**′**_septal/lateral/mean_=ratio of E to e**′**_septal/lateral/mean_ | | |
